# Dietary changes needed to reach nutritional adequacy without increasing diet cost according to income: An analysis among French adults

**DOI:** 10.1371/journal.pone.0174679

**Published:** 2017-03-30

**Authors:** Matthieu Maillot, Florent Vieux, Fabien Delaere, Anne Lluch, Nicole Darmon

**Affiliations:** 1 MS-Nutrition, Faculté de Médecine La Timone, Marseille, France; 2 Danone Nutricia Research, Centre Daniel Carasso, Palaiseau, France; 3 NORT, Aix-Marseille Université, INRA, INSERM, Marseille, France; University of Cambridge, UNITED KINGDOM

## Abstract

**Objective:**

To explore the dietary changes needed to achieve nutritional adequacy across income levels at constant energy and diet cost.

**Materials and methods:**

Individual diet modelling was used to design iso-caloric, nutritionally adequate optimised diets for each observed diet in a sample of adult normo-reporters aged ≥20 years (*n* = 1,719) from the Individual and National Dietary Survey (INCA2), 2006–2007. Diet cost was estimated from mean national food prices (2006–2007). A first set of free-cost models explored the impact of optimisation on the variation of diet cost. A second set of iso-cost models explored the dietary changes induced by the optimisation with cost set equal to the observed one. Analyses of dietary changes were conducted by income quintiles, adjusting for energy intake, sociodemographic and socioeconomic variables, and smoking status.

**Results:**

The cost of observed diets increased with increasing income quintiles. In free-cost models, the optimisation increased diet cost on average (+0.22 ± 1.03 euros/d) and within each income quintile, with no significant difference between quintiles, but with systematic increases for observed costs lower than 3.85 euros/d. In iso-cost models, it was possible to design nutritionally adequate diets whatever the initial observed cost. On average, the optimisation at iso-cost increased fruits and vegetables (+171 g/day), starchy foods (+121 g/d), water and beverages (+91 g/d), and dairy products (+20 g/d), and decreased the other food groups (e.g. mixed dishes and salted snacks), leading to increased total diet weight (+300 g/d). Those changes were mostly similar across income quintiles, but lower-income individuals needed to introduce significantly more fruit and vegetables than higher-income ones.

**Conclusions:**

In France, the dietary changes needed to reach nutritional adequacy without increasing cost are similar regardless of income, but may be more difficult to implement when the budget for food is lower than 3.85 euros/d.

## Introduction

In spite of a global improvement in living conditions, Europe still shows social inequalities in health, with differences in morbidity and mortality according to socioeconomic position (SEP)[1–3]. These inequalities are particularly significant in France, and have been widening [4]. Nutrition, a major determinant of health, greatly contributes to these inequalities[5] with unhealthy eating increasing the risk not only of obesity, but also of nutritional deficiencies and chronic diseases such as diabetes, hypertension and cardiovascular diseases[6].

Food choices are influenced by individual, cultural, social, economic and environmental factors [7–9]. Unhealthy dietary patterns are not exclusive to individuals with a low SEP, but are more frequent in this population than in wealthier population strata[10–14]. Foods of lower nutritional value and lower-quality diets generally cost less per calorie. They are more often consumed by groups of lower SEP[15], probably because individuals with limited resources are more strongly influenced by prices when they purchase food[16–19]. Given that food expenditure accounts for a larger share of a low-income household’s budget[20,21], and that cost constraints orient food choices towards less healthy foods[22,23], economic constraints are held to contribute to the higher prevalence of unhealthy eating among individuals with low SEP[15].

International official bodies encourage the integration of the multiple determinants of food choices in the development of food-based dietary guidelines[24–26]. To help fight socioeconomic inequalities in health, economic factors should receive particular attention. However, methodological advice for designing affordable healthy diets is generally not provided in food-based dietary guidelines. In the United States, diet optimisation techniques have been implemented to generate a series of affordable nutritious diets, the “Thrifty Food Plan”, used to set up the Supplemental Nutrition Assistance Program[27]. In France, foods with a very good nutritional quality/price ratio, i.e. foods able to provide a diet that meets recommendations for minimal cost, were identified using diet modelling[28], but the cultural and social acceptability of lowest cost nutritious diets has been questioned[29,30]. To address this issue, individual diet modelling techniques able to integrate not only cost and nutrition considerations, but also individual food patterns and preferences[31] have been developed.

Based on data from a French national dietary survey and mean national food prices, the present study explored the relationship between income, diet quality and diet cost. Using individual diet modelling techniques, the study then investigated whether the dietary changes needed to reach nutritional adequacy at the individual level at constant energy and diet cost were dependent on income.

## Methods

### Population sample and survey protocol

The French Individual and National Dietary Survey (INCA2) survey was conducted in 2006–2007 by the French Agency for Food, Environmental and Occupational Health Safety (ANSES) to assess dietary intake and associated behaviours in a nationally representative sample of French people[32]. The survey included two home visits by trained investigators. Socioeconomic, demographic, and behavioural variables were collected from individuals using a self-administered questionnaire and an interview. During the first visit, the investigator spent 45–60 minutes explaining the survey and food record. After seven days, the investigator returned to review both documents (e.g. to check for often forgotten foods such as bread or water in the food record, and whether there were any questions missed in the self-administered questionnaire). The investigator then conducted an interview regarding socioeconomic status and lifestyle. The multi-stage cluster sampling technique is described elsewhere[32]. To ensure national representativeness, each individual was assigned a weighting factor for unequal sampling probabilities and for differential non-responses.

In the present study, young persons between adolescence and adulthood (i.e., *n* = 72 persons aged less than 20 years) were excluded from the initial INCA2 sample (*n* = 2,624), leaving a sample of 2552 adults aged 20–79 years (S1 Fig). Under-reporters were then removed using the Black equations[33], leaving a sample of 1726 adults. Diet optimisation was not mathematically feasible in seven individuals. The present analyses were thus conducted on a final sample of 1719 individuals (hereafter “study sample”) for which it was possible to design an optimised diet with the ID models.

### Ethics

For practical reasons, recruitment of participants was by phone contact, and oral consent was obtained during the call. All procedures involving human subjects/patients were approved by the French Data Protection Authority (Commission Nationale Informatique et Libertés). The INCA2 study was conducted in accordance with the guidelines laid down in the Declaration of Helsinki.

### Sociodemographic and socioeconomic variables

Gender, age, marital status (single or couple), number of children, educational level, socio-occupational status and current smoking status were available for each participant. Level of education was ranked “high” (university level and equivalent), “intermediate” (high school), and “low” (mid-secondary or under). Socio-occupational status was ranked “high,” “intermediate”, and “low”. “High” was assigned to executive, top-management and professional classes, “intermediate” to middle professions (office employees, technicians, and similar), and “low” to manual workers and unemployed persons. A fourth class, labelled “others”, comprised retired persons, students and spouses not available for employment[32].

### Income per consumption unit

A socioeconomic questionnaire, including total household income and number of individuals living in the household, was submitted to each participant. Some missing values (20%) were reported for household income. In this case, to estimate household incomes, individuals with missing information were matched to individuals with complete income data according to their sociodemographic (age, gender, marital status and number of children) and socioeconomic (socio-occupational status, educational level) characteristics, and living standard variables (home owner or not, home equipment) using the Kohonen algorithm[34,35].

Income per consumption unit (hereafter “income”) was then calculated as self-reported household total net income divided by the number of consumption units in the household. The number of consumption units was calculated using the Organization for Economic Cooperation and Development modified equivalent scale (1 consumption unit for the householder, 0.5 for other household members aged 14 or over and 0.3 for each child under 14)[36].

### Dietary data

Dietary intake was assessed using a 7-day food record on the study sample of 1719 adults. The foods declared as consumed by the participants (*n* = 1,314 foods and non-alcoholic beverages, including water) were placed in 9 food groups, 25 food sub-groups and 54 food categories in the national food nutrient composition database (CIQUAL 2013 [37]) associated with the survey. Food group and sub-group quantities, and energy and nutrient contents (macronutrients, sodium, free sugars, saturated fatty acids, essential fatty acids, fibre, vitamins and minerals) were calculated for each individual diet.

### Diet cost

Diet cost was calculated by multiplying the quantity of each food in the diet by its mean national price. Diet cost was expressed per day or per 2,000 kcal (i.e. energy cost). As previously described[34], mean national prices were expressed in euros per 100 g of edible food, and were obtained beforehand from the 2006 Kantar-World Panel purchase database[38], which gives the annual food expenditures of a representative sample of 12,000 French households. Briefly, for each food in the food composition database, mean price was first calculated, in euros per 100 g of food as purchased, by dividing annual expenditure by the amounts purchased for all the food products corresponding to this item in the Kantar-World Panel database. Given that these prices were paid by a representative panel of consumers, the mean national prices were more likely to represent the most frequently purchased forms of each food. The prices were then expressed in euros per 100 g of edible food, using the appropriate conversion factors [37] to take into account trimming and cooking.

### Indicators of nutritional quality

Solid energy density (SED), mean adequacy ratio (MAR) and mean excess ratio (MER) were used as indicators of nutritional quality, and were estimated for each individual observed diet. SED, in kcal/100 g, was calculated based on items typically consumed as *foods*, including soups, but excluding drinking water and items typically consumed as *beverages*, such as milk, juices and other drinks[39]. SED was calculated by dividing total energy provided by solid foods by the weight of solid foods. A high SED is associated with low diet quality[40]. MAR was used as an indicator of good nutritional quality, and was calculated for each individual observed diet as the mean percentage of daily recommended intakes for 20 key nutrients (proteins, fibre, retinol equivalents, thiamine, riboflavin, niacin, vitamin B6, folates, vitamin B12, ascorbic acid, vitamin E, vitamin D, calcium, potassium, iron, magnesium, zinc, copper, iodine, and selenium), as previously described[34]. MER, an indicator of bad nutritional quality, was calculated as the average daily percent excess of sodium, saturated fatty acids and free sugars, as proposed by Vieux[41]. In the MAR and MER calculations, a high (low) intake of one component could not compensate for the low (high) intake of another. Energy cost in euros per 2000 kcal (estimated as described above) was also considered as an indicator of nutritional quality[42].

### Diet modelling

Nutritionally adequate iso-caloric diets were designed with an upgraded version of the previously described Individual Diet models (ID models)[31]. Models were built based on the assumption that individuals prefer to eat what they are used to eating, and are not willing to change their energy intake[43]. For each individual in the final study sample, a new modelled diet was designed that stayed as close as possible to the observed diet, while simultaneously meeting all nutrient recommendations at the same energy level. In the seven diets for which optimisation was not feasible (i.e., those individuals excluded from the final sample) the infeasibility was due to an incompatibility between the iso-caloric constraint and the constraints on proteins, carbohydrates and fats.

Linear programming models are typically defined by a list of decision variables, an objective function and a set of constraints[44].

**Fig 1:**
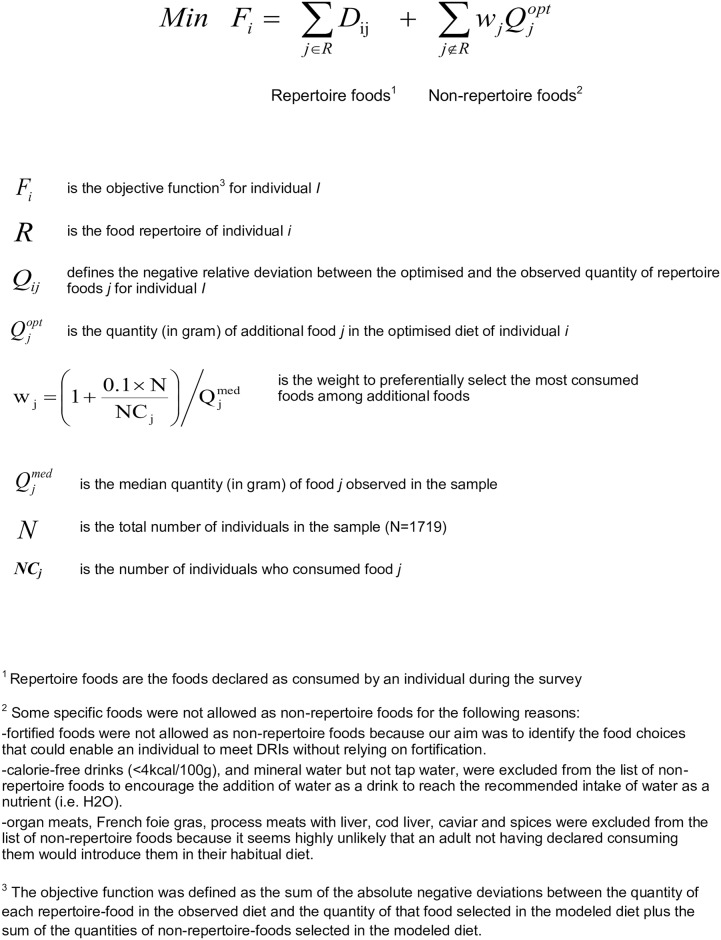
Description of the objective function.

The decision variables were the amounts of foods available for diet modelling (Fig 1). These food variables were divided into repertoire foods (foods declared as consumed by the individual) and non-repertoire foods (foods declared as consumed at least once in the survey, but not by this individual).

The objective function was defined to find the modelled diet that came as close as possible to the corresponding observed diet. As previously reported[31], the objective function aimed at (i) preferentially choosing repertoire foods, (ii) minimising the reduction of the repertoire foods and (iii) introducing non-repertoire foods (i.e. foods that were not declared as consumed). Regarding the introduction of non-repertoire foods, to favour acceptability, the foods most frequently eaten by the French population were preferentially selected, and they were introduced in the lowest quantity possible. The rationale behind the construction of this objective function was to propose a diet in which the consumption of individually preferred foods is encouraged whenever possible, while the introduction of new foods is limited as much as possible. To do this, reductions in consumed quantities of foods were penalised, but not increases. Because we did not know which foods would actually be preferred by each individual, we assumed that the foods most frequently consumed by the population would be the most likely to be accepted as new foods.

The constraints to be met were a set of nutritional constraints based on dietary reference intakes (DRIs) [45][46][47][48], a set of acceptability constraints (e.g. maximum amounts of foods and food groups) and a set of other constraints, in particular total diet weight and total diet cost (Table 1). Calorie-free drinks were excluded from the calculation of total diet weight to avoid competition between calorie-free drinks and nutrient-dense foods with low energy content.

**Table 1:** Constraints used in the individual diet models.

Constraint	Target
**Nutrients constraints**	
Energy, kcal/d*	= Observed Intake (OI)†
H_2_O g/d	≥ 2500‡
Proteins§, g/kg/d	≥ 0.83
Total fats§, % energy	20–35
Carbohydrates§, % energy	50–75
Cholesterol§, mg/d	≤ 300 or OI ||
Alpha-linolenic acid§, % energy	≥ 0.5
Linoleic acid§, % energy	2.5–9
DHA plus EPA§, g/d	≥ 0.25
Omega 3§, % energy	0.5–2
PUFA§, % energy	6–11
SFA§, % energy	≤ 10 or OI ||
Free sugars§, % energy	≤ 10 or OI ||
Sodium¶, mg/d	1500–2759 or OI ||
Fibre, 10 vitamins, 9 minerals**	≥ EAR or OI or RDA††
**Food group constraints**	
Food groups	≤ p95‡‡ or OI §§
Food sub-groups	≤ p95‡‡ or OI §§
Food categories	≤ p95‡‡ or OI §§
Individual foods	≤ p95‡‡ or OI §§
**Other constraints**	
Total diet weight, in g/d	≤ 115% of OI ||||
Diet cost, euros/d	Free or = OI depending on the model used

*1 kcal = 4·184 kJ

^†^ OI = Observed Intake

^‡^Scientific opinion of the EFSA on the Adequate Intake for water as a nutrient, i.e. H_2_O: 2500 g was the minimal daily amount recommended for men, 2000 g for women[45]

^§^WHO guidelines were used for proteins[46], fats including total fats, carbohydrates, cholesterol, ALA, LA, DHA plus EPA, Omega 3, PUFA, SFA and free sugars[6]

^||^ OI was used as an upper bound to avoid deterioration of the observed diet

^¶^ Nordic Nutrient Recommendations[47]: 2759 mg (i.e. 7 g NaCl) was the upper bound for men, 2365 mg (i.e. 6 g NaCl) for women.

** French RDAs were used for fibre, vitamin A, thiamine, riboflavin, niacin, pantothenic acid, vitamin B6, folates, vitamin B12, ascorbic acid and vitamin E, and for calcium, phosphorus, potassium, iron, magnesium, zinc, copper, iodine and selenium[48]

^††^ Constraints took into account the age and gender of each individual, and the level of the OI for each nutrient. The minimum levels imposed were as follows: at least the Estimated Average Requirement (EAR, set at 77% of RDA values) when OI was lower than EAR, at least the Recommended Dietary Allowance (RDA) when OI was greater than RDA, and equal to OI when it lay between EAR and RDA [31] Values of French RDA used in this study for the 10 vitamins and the 9 minerals were previously published [31]. A value of 30 g/d was applied for fibre

^‡‡^ P95 means 95th percentile and was calculated among consumers only and by gender

^§§^ OI was used as upper bound to take into account the specific features of individual food choice

^||||^ Excluding calorie-free drinks containing less than 4 kcal per 100 g and all kinds of water, to avoid competition between calorie-free drinks and nutrient-dense foods with low energy content

As previously described, all the modelled diets were iso-caloric with the corresponding observed ones to identify the minimal dietary changes needed to reach nutritional adequacy at constant energy. To improve the relevance of individual diet modelling, some changes were made to the previously published ID models[31]. All the changes made to the original ID models[31] are described in the S1 Method. A first set of ID models (hereafter “free-cost ID models”) was run without a cost constraint to explore the impact of the optimisation process on diet cost. A second set of ID models (hereafter “iso-cost ID models”), was then run by introducing a cost constraint in the models to set the cost of the optimised diet equal to that of the corresponding observed diet.

### Statistical analysis

Income quintiles (Q1 the lowest, Q5 the highest) were defined on the initial sample (*n* = 2,624), using income per consumption unit as the income variable. The sociodemographic and socioeconomic variables of individuals in the study sample (*n* = 1,719) were described across income quintiles, together with food and nutrient characteristics of observed diets.

For diets optimised without a cost constraint, the average variation of diet cost between the observed and optimised diets was analysed in the overall study sample, and by income quintile.

For diets optimised with the equality constraint on cost, the dietary changes between observed and optimised diets were described by food group and food sub-group for the overall study sample and by income quintile.

The nonparametric chi-squared test was used to compare the distribution of gender and other socioeconomic variables across income quintiles. Adjusted GLM models accounting for survey design were used to test the significance of income effects on both observed diets and the dietary changes induced by the ID models to reach nutrient adequacy. As strong gender-based patterning effects of social gradient in diets has been found in some populations [49] a gender*income quintiles interaction term was tested in all the analyses as a sensitivity analysis.

Energy intake, age, gender, marital status and number of children were used as a first set of adjustment variables. In a second set socio-occupational status, educational level and current smoking status were added as covariables. When the income effect was significant, a linear trend was also tested (*p* for trend). An additional analysis, based on GLM and non-parametric chi-squared test accounting for survey design was conducted to compare the sociodemographic and socioeconomic characteristics of individuals in the final study sample (*n* = 1,719) with those excluded from the analysis (*n* = 905).

The Operational Research and STAT packages of SAS version 9.4 (SAS institute, Cary, NC) were used to run linear programming models and perform statistical analysis, respectively. To ensure robustness in conclusions, a conservative approach was used, with an alpha level of 1% for all the statistical tests.

## Results

### Sociodemographic and socioeconomic characteristics of the sample, by income quintile

Income quintiles were positively associated with the two other socio-economic variables, namely socio-occupational status and educational level (S1 Table). Age significantly increased across income quintiles. There was also a significant relation between income quintiles and marital status, with more single individuals in the lowest income quintile. Individuals in the lowest income quintile were more likely to be women, although the overall association across income quintiles was not significant. Compared with the final study sample, the excluded individuals were significantly more likely to be younger, have a lower income, live alone, have a lower educational level and a lower socio-occupational status, and be current smokers (see S2 Table).

### Nutrient and food characteristics of observed diets, by income quintile

In the study sample, the average energy intake was not significantly different according to income quintiles (Table 2). The MAR score, daily diet cost and energy cost of observed diets were significantly and positively associated to income quintiles (significant *p* value of trend with adjustment for energy intake, gender, age, marital status and number of children, and they remained significant after additional adjustments (current smoking status, educational level and socio-occupational status). In particular, MAR increased from 81.9% to 84.8% adequacy/d from Q1 to Q5. Diet cost increased from 6.4 to 7.2 euros/d across income quintiles. Total diet weight, macronutrient energy contributions, solid energy density, MER score and its components (sodium, free sugars and saturated fatty acids), were not significantly different according to income quintiles.

**Table 2:** Characteristics of observed diets in the overall study sample (*n* = 1,719) and by income quintile (Q1 lowest, Q5 highest).

	All		Income quintile from lowest (Q1) to highest (Q5)											Adjusted *p-* value*
	(*n* = 1719)		Q1 (*n* = 330)		Q2 (*n* = 305)		Q3 (*n* = 375)		Q4 (*n* = 385)		Q5 (*n* = 324)			
	Mean#	SD	Mean	SD	Mean	SD	Mean	SD	Mean	SD	Mean	SD	*p*_1_†	*p*_2_‡
Energy intake||¶ (kcal/d)	2151.0	535.9	2119.3	573.6	2144.2	500.1	2169.8	530.0	2185.6	567.3	2126.8	495.4	-	-
Proteins (% energy)	16.5	2.7	16.6	3.1	16.6	2.8	16.5	2.5	16.3	2.4	16.4	2.6	0.140	0.465
Carbohydrates (% energy)	42.7	6.1	43.4	6.6	43.1	5.9	42.3	5.7	42.7	6.1	42.2	6.3	0.142	0.077
Fats (% energy)	38.5	5.7	37.8	5.8	38.0	5.7	38.9	5.2	38.6	5.8	38.9	5.8	0.079	0.063
Total diet weight (g/d)	2622.4	745.8	2551.7	850.2	2568.9	749.0	2632.0	675.2	2687.0	731.6	2660.7	718.0	0.133	0.578
SED** (kcal/100g)	173.4	32.7	175.5	34.6	173.1	31.3	176.5	32.6	171.3	32.4	170.4	32.0	0.187	0.292
MAR score (% adequacy/d)	83.8	9.0	81.9	10.1	83.5	9.4	83.6	8.8	85.1	8.4	84.8	8.0	<0.001	<0.001
MER score (% excess/d)	32.2	30.0	32.4	34.3	31.3	26.9	33.7	29.0	33.1	32.0	30.1	26.5	0.282	0.515
Na (mg/d)	3061.2	968.4	2956.5	1000.4	3116.5	969.1	3077.3	952.6	3133.1	1017.7	3012.0	882.9	0.834	0.644
Free sugars (% energy)	9.5	5.1	10.2	6.0	9.3	5.3	9.5	4.8	9.3	4.7	9.3	4.5	0.171	0.027
Saturated fatty acids (% energy)	14.7	3.0	14.4	3.3	14.5	3.0	15.0	2.8	14.6	3.0	14.8	3.0	0.157	0.069
Diet cost (euros/d)	6.8	1.8	6.4	1.8	6.6	1.7	6.8	1.8	7.1	1.8	7.2	1.7	<0.001	<0.001
Energy cost (euros/2000kcal)	6.5	1.4	6.1	1.3	6.3	1.3	6.4	1.5	6.7	1.3	6.9	1.5	<0.001	<0.001

^#^ all means are survey-weighted

* Significant differences between quintiles for an alpha level of 1%

^†^ Adjusted for energy intake, age, gender, marital status, number of children

^‡^ Adjusted for energy intake, age, gender, marital status, number of children, current smoking status, educational level and socio-occupational status

^||^ Energy intake was not significantly different according to income quintile (unadjusted *p* = 0.428)

^¶^ 1 kcal = 4·184 kJ

** SED, solid energy density, calculated based on items typically consumed as *foods*, including soups, but excluding drinking water and items typically consumed as *beverages*, such as milk, juices and other drinks

Very few significant differences were found across income quintiles regarding food intakes (Table 3). For some sub-groups, namely vegetables and nuts, the differences were significant with the first set of adjustments, but did not remain so with the full adjustment set. By contrast, for the starchy foods group and for the fruit and vegetables group, and for the fruit sub-group, the differences were significant only with the full set of adjustments. Intakes in the fruit and vegetables food group thus increased from 342 g/d to 422 g/d (i.e., a difference of 1 portion of 80 g/d) between Q1 and Q5, mainly due to an increase in fruit consumption (from 137 to 195 g/d). The sensitivity analysis showed a significant gender-income interaction only for hot drinks, with no strong tendencies across quintiles.

**Table 3:** Observed food group and subgroup intakes (g/d) for the overall study sample (*n* = 1,719) and by income quintile (Q1 lowest, Q5 highest).

	All		Income quintile from lowest (Q1) to highest (Q5)										Adjusted *p-*value*	
	(*n* = 1719)		Q1 (*n* = 330)		Q2 (*n* = 305)		Q3 (*n* = 375)		Q4 (*n* = 385)		Q5 (*n* = 324)			
	Mean#	SD	Mean	SD	Mean	SD	Mean	SD	Mean	SD	Mean	SD	*p*_1_†	*p*_2_ ‡
Fruit and vegetables	379.7	236.7	342.4	220.3	366.8	223.4	349.3	227.5	417.4	257.2	422.5	238.4	0.109	0.001
Fruits	163.1	148.7	136.9	129.0	142.9	131.8	149.0	148.4	189.8	159.8	195.3	159.3	0.542	<.001
Vegetables||	214.5	142.3	203.6	141.7	222.0	140.4	198.6	134.2	225.2	156.8	224.8	133.5	0.001	0.305
Nuts¶	2.1	5.8	1.9	6.1	1.9	6.2	1.8	5.1	2.4	6.0	2.4	5.7	0.003	0.811
Starchy foods	254.2	118.9	269.9	133.6	270.4	123.4	246.5	113.9	250.4	114.0	234.9	106.1	0.056	0.010
Refined starchy	168.8	96.4	177.9	103.3	181.1	100.5	164.8	91.8	164.5	88.9	156.9	97.4	0.533	0.047
Unrefined starchy	80.5	59.3	86.9	65.9	85.8	59.2	76.4	55.4	80.3	65.4	73.5	47.4	0.176	0.084
Ready-to-eat cereals	4.9	16.3	5.1	20.0	3.6	12.0	5.3	17.3	5.6	15.4	4.5	15.2	0.012	0.365
Meats, eggs, fish	166.8	70.2	165.6	75.5	169.5	68.6	170.4	75.7	163.9	61.9	165.0	68.7	0.196	0.350
Meats**	120.4	63.9	122.0	72.7	122.6	60.9	126.4	68.7	114.8	55.8	116.3	59.6	0.011	0.090
Eggs	15.6	17.5	16.1	18.9	16.6	19.3	16.0	17.1	15.6	17.0	13.2	15.1	0.068	0.694
Fish††	30.9	29.6	27.5	29.8	30.2	27.9	28.0	28.0	33.5	29.5	35.5	32.1	0.045	0.483
Mixed dishes and salted snacks	120.8	91.4	118.8	94.1	118.6	93.3	132.0	97.3	122.8	94.0	109.5	74.4	0.410	0.369
Mixed dishes	69.9	67.7	70.4	80.2	71.2	67.7	77.5	68.6	68.1	65.4	61.6	53.2	0.587	0.455
Salted snacks‡‡	50.9	57.0	48.5	52.6	47.4	55.9	54.6	64.0	54.7	62.5	47.9	45.9	0.598	0.430
Dairy products	201.8	168.6	187.3	152.5	197.2	152.1	211.7	207.2	204.8	170.5	206.8	145.1	0.623	0.562
Milk	86.8	145.8	74.7	126.9	87.9	128.0	96.1	190.7	90.9	141.0	82.9	123.2	0.024	0.495
Fresh dairy products§§	80.9	81.3	78.1	90.6	79.7	77.7	82.0	77.6	77.7	82.4	87.8	77.3	0.162	0.753
Cheeses	34.1	28.8	34.5	34.5	29.6	25.2	33.6	31.0	36.3	26.9	36.2	24.7	0.711	0.037
Sweet products	118.4	74.6	110.6	79.1	116.6	71.3	122.3	73.2	120.3	73.2	121.8	76.2	0.011	0.898
Milk or egg-containing desserts	18.4	31.0	17.1	30.7	21.5	35.7	18.0	28.4	17.6	31.7	18.3	28.3	0.018	0.656
Cakes and Viennese pastries	65.8	52.5	57.4	52.0	64.9	52.4	71.2	53.4	68.3	50.5	66.1	53.4	0.420	0.088
Biscuits and confectionary||||	34.2	32.0	36.1	32.9	30.2	30.7	33.1	32.1	34.4	29.8	37.4	34.2	0.492	0.077
Water and beverages	1331.6	631.2	1307.8	722.4	1281.2	619.2	1351.4	577.4	1357.2	636.2	1351.6	595.7	0.192	0.818
Water	798.5	569.3	747.0	573.2	780.0	575.9	820.6	564.6	824.5	581.2	814.5	549.3	0.755	0.824
Hot drinks	396.8	333.3	399.0	412.1	365.9	290.0	393.1	300.1	401.3	324.3	424.0	328.1	0.008	0.775
Diet sweet beverages	12.9	62.2	14.5	55.5	10.7	53.6	15.1	68.8	10.4	50.3	14.0	78.8	0.348	0.537
Sugar-sweetened beverages	64.0	176.3	92.0	203.7	71.6	228.9	63.9	152.4	53.4	143.7	38.7	143.8	0.946	0.281
Fruit juices 100%	59.3	90.0	55.2	97.4	52.9	80.8	58.7	85.8	67.5	104.7	60.5	74.4	0.311	0.311
Added fats and sauces	45.5	23.4	44.8	25.0	45.3	22.8	45.7	22.9	46.6	25.3	45.0	20.6	0.847	0.914
Animal fats	14.2	13.6	13.6	13.7	14.6	13.4	15.4	15.3	13.2	12.5	14.1	12.8	0.960	0.350
Vegetable fats	23.9	16.3	23.4	16.1	23.7	16.5	23.1	16.3	25.3	17.6	23.8	14.9	0.966	0.853
Spices and aromatic plants	7.4	10.9	7.8	11.5	7.0	8.8	7.1	9.5	8.0	14.2	7.1	8.6	0.109	0.777
Foods based on soya	3.5	25.4	4.5	31.8	3.3	26.0	2.6	23.5	3.7	18.7	3.5	26.9	0.542	0.947

^#^ all means are survey-weighted

* Significant differences between quintiles for an alpha level of 1%

^†^ Adjusted for energy, age, gender, marital status, number of children

^‡^ Adjusted for energy intake, age, gender, marital status, number of children, current smoking status, educational level and socio-occupational status

^||^ Including soups

^¶^ Including dried fruits

** Including organ meats

^††^ Including sea products

^‡‡^ Including sandwiches

^§§^ Including yogurts, fermented milks and associated French specialities (“fromage blanc” and “petit-suisse”)

^||||^Including chocolate and sugar

### Impact of the free-cost ID models on diet cost, by income quintile

**Fig 2:**
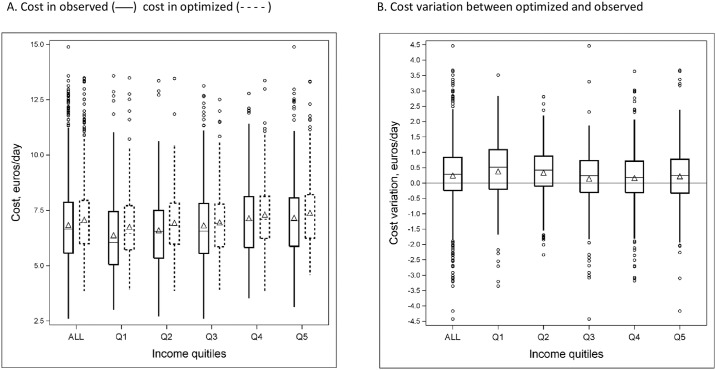
Impact of the optimisation with the free-cost ID models on diet cost in the overall study sample (*n* = 1719) and by income quintile. (A) Distributions (minimum, 1st quartile, median, 3rd quartile and maximum) and average values (triangle symbols) of diet cost (in euros/d) in observed and optimised diets. In each income quintile and for the overall study sample, diet cost distribution was significantly different between observed and optimized diets, whatever the adjustments. (B) Distributions of the cost variations between optimised and observed diets. Mean cost variation in the overall sample (*n* = 1,719) was significantly different from zero (*p* < 0.01) whatever the adjustments. Mean cost variations were not significantly different across income quintiles (*p* = 0.094), whatever the adjustments.

The distributions of diet cost in observed and optimised diets, and the variations in cost induced by the optimisation process are shown in Fig 2. In the overall study sample, the lowest diet cost was 2.60 euros/d in observed diets against 3.85 euros/d in the optimised diets (Fig 2A). In other words, whatever the income quintile, when cost was not constrained, the optimisation process systematically increased diet cost when it was lower than 3.85 euros/d in the observed diet. Diet cost was increased on average by +0.22 euros/d (corresponding to +3.2% of the mean observed diet cost). This significant increase was maintained within each income quintile, whatever the adjustments (Fig 2B). For most of the individuals (64%) daily diet cost increased in order to reach nutritional adequacy, and this proportion was unequally distributed across income quintiles (67.9%, 71.8%, 61.9%, 60.6%, 59.3% from Q1 to Q5 respectively, data not shown). The variation in diet cost between observed and optimised diets was not significantly different across income quintiles. Diet cost difference between observed and optimised diets ranged widely (extremes were −5.06 and +4.46 euros/d).

### Dietary changes induced by the iso-cost ID models, by income quintile

Introducing the cost constraint did not alter the feasibility of the ID models: it was feasible to design an optimised diet at no extra cost for each individual in the study sample.

On average, diet optimisation increased diet weight by 300 g/d, with 137 g from repertoire foods and 163 g from non-repertoire added foods (Table 4). The increase in total diet weight was tendentially greater for lower income quintiles, but the difference did not reach significance after additional adjustments. On average, 7.7 repertoire foods were removed from an individual diet, and 4.5 non-repertoire foods were added. The number and weight of non-repertoire foods added by the optimisation process significantly increased with decreasing income quintiles, but significance was lost with additional adjustments.

**Table 4:** Food weight variations and repertoire food changes induced by the iso-cost ID models for the overall study sample(*n* = 1,719) and by income quintile (Q1 lowest, Q5 highest).

	All		Income quintile from lowest (Q1) to highest (Q5)										Adjusted *p*-value*	
	(*n* = 1719)		Q1 (*n* = 330)		Q2 (*n* = 305)		Q3 (*n* = 375)		Q4 (*n* = 385)		Q5 (*n* = 324)			
	Mean#	SD	Mean	SD	Mean	SD	Mean	SD	Mean	SD	Mean	SD	*p*_1_†	*p*_2_‡
Total diet weight (g/d)	+300.0	487.3||	+343.5	553.0	+315.0	577.9	+286.2	421.5	+276.1	430.0	+283.7	456.7	0.087	0.491
Weight of repertoire foods (g/d)	+137.3	487.9	+134.4	570.7	+121.4	547.6	+124.3	427.1	+147.4	445.7	+159.2	451.3	0.867	0.895
Weight of non-repertoire foods (g/d)	+162.7	260.6	+209.0	293.6	+193.6	312.4	+161.9	250.3	+128.7	202.0	+124.5	233.4	0.000	0.064
Number of removed repertoire foods	7.7	5.3	7.8	5.6	7.7	5.6	7.8	5.0	7.8	5.4	7.0	4.8	0.098	0.661
Number of added non-repertoire foods	4.5	4.0	5.4	4.2	4.8	4.5	4.6	3.8	3.9	3.5	3.6	3.6	<0.001	0.018

^#^ all means are survey-weighted

* Significant differences between quintiles for an alpha level of 1%

^†^ Adjusted for energy intake, age, gender, marital status, number of children

^‡^ Adjusted for energy intake, age, gender, marital status, number of children, current smoking status, educational level and socio-occupational status

^||^ All means are significantly different from zero whatever the adjustment

Dietary changes were needed to reach nutritional adequacy, and the sign of these changes was the same across all income quintiles (Table 5): at the food group level, whatever the income quintile, the optimisation increased fruits and vegetables (mostly fruits), and starchy foods (both refined and unrefined), and it decreased mixed dishes and salted snacks, and also sweet products; at the food sub-group level, other changes needed to reach nutritional adequacy were an increase in fish and a decrease in meats, an increase in milk and fresh dairy products, and a decrease in cheese, an increase in water, hot drinks and diet sweet beverages, and a decrease in sugar-sweetened beverages and fruit juices, an increase in vegetable fats and a decrease in animal fats. The only significant differences between income quintiles regarding the food changes needed to reach nutritional adequacy were for fruit (and fruit and vegetables as a food group), milk (and dairy products as a food group) and hot drinks (greater increases in Q1 *vs*. Q5), and for sugar-sweetened beverages (greater decreases in Q1 *vs*. Q5). These small differences between income quintiles were not maintained after additional adjustments, except for fruit (and the fruit and vegetables food group). The sensitivity analysis showed a significant gender-income interaction only for unrefined starchy foods, with a decreasing trend (from Q1 to Q4) among men, but not among women.

**Table 5:** Dietary changes induced by the iso-cost ID models for the overall study sample (*n* = 1,719) and by income quintile (Q1 lowest, Q5 highest).

	All			Income quintiles from lowest (Q1) to highest (Q5)										Adjusted *p*-value*	
	(*n* = 1719)			Q1 (*n* = 330)		Q2 (*n* = 305)		Q3 (*n* = 375)		Q4 (*n* = 385)		Q5 (*n* = 324)			
	Mean#	SD	*p*-value†	Mean	SD	Mean	SD	Mean	SD	Mean	SD	Mean	SD	*p*_1_‡	*p*_2_§
Fruit and vegetables	+171.1	137.7	<0.001	+178.7	137.1	+168.4	133.8	+198.0	142.4	+152.5	147.8	+156.2	117.8	<0.001	0.001
Fruits	+151.9	105	<0.001	+149.3	109.9	+160.3	108.7	+167.8	105.6	+143.7	101.7	+137.9	96.9	0.001	0.003
Vegetables¶	+15.8	109.7	<0.001	+26.9	107.7	+4.9	115.4	+26.4	108.5	+5.6	117.6	+14.4	95.5	0.052	0.052
Nuts**	+3.3	6.4	<0.001	+2.5	5.3	+3.2	6.3	+3.9	7.0	+3.2	6.3	+3.9	6.7	0.058	0.176
Starchy foods	+120.6	78.7	<0.001	+117.8	82.2	+114.4	80.4	+125	78.9	+122.4	77.3	+122.4	74.6	0.678	0.769
Refined starchy	+58.2	66.9	<0.001	+53	67.5	+50.1	69.5	+64.2	66.8	+63.4	64.5	+58.1	65.8	0.098	0.183
Unrefined starchy	+62.7	47.0	<0.001	+64.9	50.5	+64.4	46.6	+61.4	45.5	+58.9	48.3	+64.8	43.5	0.461	0.436
Ready-to-eat cereals	-0.3	6.2	0.103	-0.2	7.7	-0.1	5.0	-0.6	5.8	+0.1	6.9	-0.5	4.9	0.662	0.604
Meats, eggs, fish	-28.2	50.6	<0.001	-29.4	52.4	-29.7	52.8	-31.9	55.7	-22.9	44	-27.7	47.4	0.129	0.160
Meats††	-35.1	46.2	<0.001	-37.9	51.1	-35.7	48.2	-38.6	50.8	-29.2	38.6	-34.5	41.2	0.025	0.061
Eggs	-1.6	14.5	<0.001	-1.0	14.9	-3.2	18.5	-1.7	13.9	-2.1	13.3	-0.2	11.6	0.210	0.331
Fish‡‡	+8.5	18.1	<0.001	+9.5	18.7	+9.3	17.2	+8.4	18.0	+8.4	18.3	+7	18.2	0.566	0.864
Mixed dishes and salted snacks	-51.0	67.6	<0.001	-51.3	74.4	-51.9	71.6	-58.4	67.3	-49.5	66.9	-42.7	56.3	0.165	0.796
Mixed dishes	-32.7	55.2	<0.001	-35.5	71.1	-32.2	55.6	-38.6	52.6	-30.6	50.9	-25.3	42.0	0.015	0.352
Salted snacks§§	-18.3	38.5	<0.001	-15.8	36.6	-19.7	45.4	-19.8	39.3	-18.9	36.7	-17.4	34.1	0.711	0.690
Dairy products	+20.1	104.6	<0.001	+28.7	96.4	+31.3	99.9	+10.7	132.3	+26.9	97.7	+2.2	83.9	0.004	0.053
Milk	+22.8	99.4	<0.001	+30.8	91.5	+30.7	92.1	+15.9	132.3	+29	88.5	+6.8	77.7	0.005	0.073
Fresh dairy products||||	+13.1	50.8	<0.001	+14.7	50.4	+12.8	53.9	+11	54.3	+14.8	48.1	+11.8	47.1	0.891	0.982
Cheeses	-15.8	27.5	<0.001	-16.8	34.6	-12.2	23.5	-16.2	29.6	-17.0	25.8	-16.4	21.4	0.100	0.216
Sweet products	-17.5	48.6	<0.001	-16.7	51.2	-13.2	50.5	-18.7	48.8	-18.3	46.1	-20.3	46.6	0.345	0.478
Milk or egg-containing desserts	-1.7	17.0	<0.001	-2.3	16.8	-1.2	18.3	-0.3	15.6	-2.2	16.1	-2.3	18.7	0.119	0.230
Cakes and Viennese pastries	-12.2	43.2	<0.001	-10.0	44.7	-9.1	43.2	-13.9	45.0	-12.3	39.1	-15.8	44.0	0.228	0.399
Biscuits and confectionary¶¶	-3.6	18.3	<0.001	-4.4	19.2	-2.9	16.9	-4.5	20.4	-3.8	17.2	-2.2	17.5	0.337	0.004
Water and beverages	+91.3	500.5	<0.001	+123.6	558.2	+100.3	614.9	+67.3	435.9	+73.5	441.9	+97.9	452.5	0.266	0.606
Water	+115.6	444.5	<0.001	+175.8	471.9	+98.9	540.7	+88.9	391.4	+90.3	394.8	+129.6	424.8	0.062	0.212
Hot drinks	+24.1	153.3	<0.001	+17.1	204.9	+50.0	161.3	+27.8	132.5	+22.9	126.6	+3.0	131.1	0.005	0.053
Diet sweet beverages	+4.1	41.9	<0.001	+6.6	39.0	+5.7	42.9	+2.6	30.1	+2.4	27.8	+3.6	63.8	0.295	0.313
Sugar-sweetened beverages	-39.8	137.3	<0.001	-64.6	168.8	-44.7	179.9	-41.7	117	-25.0	81.5	-23.8	125.9	0.007	0.111
Fruit juices 100%	-12.7	54.1	<0.001	-11.4	60.8	-9.6	50.5	-10.3	46.7	-17.1	61	-14.6	48.8	0.290	0.512
Added fats and sauces	-6.4	18.4	<0.001	-7.3	18.3	-6.2	18.4	-5.7	17.8	-7.8	20.4	-4.8	16.7	0.352	0.771
Animal fats	-9.5	12.2	<0.001	-9.4	12.5	-9.9	11.2	-10.5	13.7	-8.8	11.3	-9.0	11.7	0.541	0.713
Vegetable fats	+3.3	15.3	<0.001	+3.0	14.2	+3.9	14.9	+4.4	15.2	+2.3	17.6	+3.3	14.0	0.479	0.629
Spices and aromatic plants	-0.3	7.6	0.253	-0.9	6.2	-0.2	7.6	+0.4	6.2	-1.3	11.1	+0.9	4.9	0.012	0.015
Foods based on soya	+0.6	15.1	0.115	+0.5	20.8	+1.6	24.3	-0.1	6.5	+0.7	10.9	+0.4	5.6	0.497	0.550

^#^ all means are survey-weighted

* Significant differences between quintiles for an alpha level of 1%

^†^ Unadjusted *p* value of comparison to zero

^‡^ Adjusted for energy intake, age, gender, marital status, number of children

^§^ Adjusted for energy intake, age, gender, marital status, number of children, current smoking status, educational levels and socio-occupational status

^¶^ Including soups

** Including dried fruits

^††^ Including organ meats

^‡‡^ Including sea products

^§§^ Including sandwiches

^||||^ Including yogurts, fermented milks and associated French specialities (“fromage blanc” and “petit-suisse”)

^¶¶^ Including chocolate and sugar

In both sets of ID models, the most binding constraints (i.e. the constraints most difficult to meet) were those on total energy, total diet cost (when used in the model), the maximum amounts of sodium, free sugars and saturated fatty acids, and the minimum amount of total carbohydrates (data not shown).

## Discussion

In this sample of French adults, a lower income was associated with less adequate diets that were also less costly. Reaching nutritional adequacy did increase diet cost for most individuals, but by introducing an equality cost constraint, it was possible to model optimal diets without increasing diet cost, irrespective of the initial observed cost. The dietary changes induced by the optimisation were mostly similar across quintiles, although a greater fruit and vegetable increase was needed for low-income individuals to meet nutritional recommendations. Greater departure from observed diets was also needed when the budget for food was lower than 3.85 euros/d.

One strength of the present study is that income, diet quality and diet cost were concomitantly explored using data from the French dietary survey and national food prices. The results show that the diets of lower-income individuals were both less costly and less adequate than those of higher-income individuals in this French sample. Positive relationships between income (or other SEP indicator), diet cost and diet quality had previously been found in some non-representative research cohorts of adults in the UK, France and the US[50–56], and in one representative sample of adults in the US[57]. In other studies, only two out of the three dimensions (SEP, diet cost, diet quality) were analysed together. For instance, in a recent study conducted in a representative population of adults in the UK, a positive relation between having a lower income and consuming a diet of lower monetary value was also found, but no attempt was made to concomitantly assess the nutritional quality of the diets[58]. In line with previous studies that have explored the relationship between diet cost and dietary quality[11,13,50,59,60], we also found a positive link between those two dimensions. The present results are also consistent with those of several studies conducted in industrialized countries[10,11], including France[7,61], that have found a positive relationship between SEP and dietary quality. In the present study, there were very few differences across income quintiles regarding food intakes. For fruit and vegetables however, persons in the lowest income quintile consumed on average one portion of 80 g less than those in the highest quintile, which may contribute to the lower nutrient adequacy (i.e. lower MAR) of their diets. By contrast, energy and macronutrient intakes were not linked to income levels, nor were the intakes of sodium, free sugars and saturated fatty acids, and consequently MER. The observation that SEP affected the positive aspects of the diet more than the negative ones, except perhaps the consumption of sweet beverages[12,34,62], is also consistent with previous findings, including in the US population[63,64].

Another strength of this study is that for each individual in the population, we were able to identify the smallest dietary changes needed to meet the full set of nutrient recommendations without increasing cost. Compared with the observed diets, optimisation with the ID models increased all plant-based foods, and it decreased almost all animal-based foods except fish and milk and fresh dairy products, showing general compliance with current dietary guidelines[65–67]. An increase in hot drinks and a decrease in sugar-sweetened beverages and fruit juices were also induced by the ID models. All these optimisation-driven dietary changes were consistent with previously published results obtained with ID models with no cost constraint[31,68]. They were also highly consistent between income quintiles.

This is the first time that the cost dimension has been explored using individual diet modelling. In the absence of cost constraint, diet cost increased by 0.22€/d on average to reach nutritional adequacy, confirming the known positive link between diet quality and diet cost[11,13,50,59,60]. However, even though diet cost increased on average, it also decreased in some cases: within each income quintile, the variation in cost induced by the ID models with no cost constraint was highly variable, and was not significantly different across income quintiles. This recalls studies in the US, where a high variability of nutritional quality was observed within each diet cost quintile[60], and conversely, a high variability of diet cost was found within each quintile of diet quality[69], showing the role of factors other than economic in determining dietary choices and nutritional quality of diets.

The lowest cost was 2.60 euros/d for observed diets, but 3.85 euros/d when diets were optimized in the absence of cost constraint, showing that for individuals with an observed diet cost lower than 3.85 euros/d, a greater departure from their habitual dietary pattern would be needed to reach similar nutritional goals than for the rest of the population. Nevertheless, when the optimisation was conducted at iso-cost, it was possible to model a nutritionally adequate diet whatever the initial cost, even the lowest one, i.e. 2.60 €/d. This apparently paradoxical finding is in line with a study based on population diet modelling where the minimal cost needed to achieve a nutritious diet for French adults was estimated at 3.50 €/d, but could be as low as 1.50€/d when substantial deviations from habitual food consumption patterns were tolerated[30]. Similarly, in the present study, it was possible to obtain a nutritious diet at very low cost, but a higher diet cost was needed to optimise the diet without too much deviation from current habits.

One important limitation is that the analyses were conducted in a sub-sample representing only 66% of the initial sample of the French adult INCA2 survey. In addition, individuals with low SEP were actually under-represented in the study sample, which may limit the generalisability of our findings. To minimise this bias, the income quintiles were calculated among the whole initial population (i.e. before the exclusions). As a result, income quintiles displayed expected relationships with most socio-demographic characteristics, as they were positively associated with socio-occupational status and educational level, with more single individuals, more women and more smokers in the lowest income quintile. Another limitation is related to dietary surveys and modelling approaches: the validity of results obtained with diet modelling analysis is dependent on the quality of input data and the relevance of the designed models. In particular, in the present study, we adopted a conservative approach based on the hypotheses that individuals, especially those with low income, were likely to maintain familiar dietary patterns within their habitual caloric intakes[70]. However, in a context where obesity rates are still rising, other models might have been more relevant, such as allowing a decrease in the energy content of modelled diets, at least for individuals identified as consuming more calories than they need, as proposed in other simulations studies[71]. The way foods were categorised can be seen as a limitation: in particular, the starchy food group did not include products based on starchy ingredients that were rich in fat, sugar and/or salt, such as cakes and biscuits (included in the sweet products food group), or burgers and crisps (included the mixed dishes and salty snacks food-group). However, this grouping is that of the French National Nutrition Programme, which recommends consuming starchy foods (e.g. bread, pasta, rice, potatoes, pulses, etc.) at each meal, while limiting the intake of sweet products and salty snacks [72]. The foods included in the starchy food group therefore had a relatively good nutrient profile [73], which may explain why both refined and unrefined starchy foods were increased (though the refined/unrefined ratio decreased) by the optimization process. Starchy foods were also increased because, among the core foods habitually consumed by the population, they are needed to meet the constraint on carbohydrates (i.e. reaching at least 50% while the observed diets averaged 42.7%). In line with previous studies [68], the carbohydrate constraint was identified as one of the most difficult constraints to meet, together with the constraints on the maximum amounts of sodium, free sugars and saturated fatty acids. Also, modelled diets were generated regardless of the usual combinations between foods (e.g. bread and butter for toast), and some normally combined foods could be individually removed (e.g. butter), undermining the realism of a few optimised diets. Nevertheless, diet modelling is well-recognized by the scientific community as a flexible, robust approach to translating nutrient recommendations into realistic food choices[74]. Another limitation is the use of mean food prices instead of real food expenditure to estimate diet cost. It assumes that prices are constant, ignoring variations in time and space. However, in the present study, the food price database and dietary intake database were concurrent (2005–2006), and so we can assume that price variations due to inflation were contained. In addition, the use of mean food prices may underestimate the food budget of wealthier individuals and overestimate that of low SEP. For instance, the real expenditure for food of low income French individuals is 1€/person per day lower than when estimated with mean national prices[75]. Hence a calculation based on average food prices is only a proxy for real expenditure and actual diet cost. In particular, it does not integrate food purchasing strategies, such as buying lower-cost options for the same food item. However, one strength of estimating diet cost with mean prices is that variations of estimated diet cost do reflect actual differences in terms of dietary choices, and can be considered as a marker for the quality of food choices. Finally, to our knowledge, no large-scale studies have measured both individual food consumption and actual expenditure on those foods. We are aware that both dietary data from INCA2 and food price data were almost ten years old, but they were the most recent open data available for the French population at the time of the study. Although merging individual dietary data with average price databases was the best tool available for diet cost analysis at the time[15], further studies exploring actual food purchases merged with nutritional composition of real food products are now needed.

In conclusion, the present study shows that to improve diet quality, dietary recommendations can be roughly similar in terms of food groups and sub-groups for all individuals whatever their income. However, the results suggest that to reach nutritional adequacy without changing energy intake, low-income individuals would have either to adopt costlier food choices or to accept wider deviations from their habitual diet.

These results support providing fruit and vegetable vouchers for low-income persons [76], and suggest that in France the target population of such a public health measure could be identified by a food budget lower than 3.85 euros/d.

## Supporting information

S1 Fig Sample flowchart. (DOCX)

S1 Method List of changes made to the previously published individual diet models. (DOCX)

S1 Table Sociodemographic characteristics of individuals in the study sample (All) and by income quintile (Q1 lowest, Q5 highest). (DOCX)

S2 Table Sociodemographic characteristics of individuals in the initial INCA2^1^ adult sample (*n* = 2,624), in the sample of excluded individuals^2^ (*n* = 905) and in the final study sample (*n* = 1,719). (DOCX)
