# Supplementary material for: Dietary changes needed to reach nutritional adequacy without increasing diet cost according to income: An analysis among French adults
Source: PLoS One. 2017 Mar 30;12(3):e0174679. doi: 10.1371/journal.pone.0174679 (PMC5373615; doi:10.1371/journal.pone.0174679)
Supplement: S1 Fig — (DOCX) [file pone.0174679.s001.docx]

**S1 Fig. Sample flowchart**
