# Supplementary material for: Dietary changes needed to reach nutritional adequacy without increasing diet cost according to income: An analysis among French adults
Source: PLoS One. 2017 Mar 30;12(3):e0174679. doi: 10.1371/journal.pone.0174679 (PMC5373615; doi:10.1371/journal.pone.0174679)
Supplement: S1 Method — (DOCX) [file pone.0174679.s002.docx]

**S1 Methods. List of changes made to the previously published Individual Diet models** (1)

*Nutritional constraints*

Sodium, free sugars and saturated fatty acids were limited to the maximal recommended values or to the observed intakes when they were lower than the maximal recommended values. A constraint imposing a minimal amount of water as a nutrient (i.e., H_2_O) was added and was set to the EFSA opinion adequate intake (at least 2000g/d for women and at least 2500g/d for men) (EFSA, 2010) (2).

*Food variables*

The list of variables was extended to tea, coffee and drinking water. However, calorie-free drinks (<4kcal/100g) and all mineral water but not tap water were removed from the non-repertoire food variables to encourage the addition of tap water in order to reach the recommended intake of water as a nutrient (i.e., H_2_O).

*Objective function*

To limit the increase of fortified foods and to reach the minimal requirement of H_2_O intake by selecting primarily tap water, positive deviations of fortified foods (i.e. ready to eat cereals) and of hot drinks (tea, coffee and hot chocolate) were took into account in the minimization. The weighing coefficients of non-repertoire foods were also modified to further penalize those foods consumed by less than 10% of individuals.

*Total weight constraint*

As previously described, the total weight of the modeled diet was limited to 115% of total diet weight of the observed diet (1). However, in this upgraded version, calorie-free drinks were excluded from the calculation of total diet weight. This decision was made to avoid the competition between calorie-free drinks and nutrient dense foods with low energy content.

***References***

1. Maillot M, Vieux F, Amiot MJ, Darmon N. Individual diet modeling translates nutrient recommendations into realistic and individual-specific food choices. Am J Clin Nutr [Internet]. 2010;91:421–30. Available from: <Go to ISI>://WOS:000273947500018

2. EFSA Panel on Dietetic Products Nutrition and Allergies (NDA). Scientific Opinion on Dietary Reference Values for water. EFSA J 2010. 2010;8.
