# Supplementary material for: Dietary changes needed to reach nutritional adequacy without increasing diet cost according to income: An analysis among French adults
Source: PLoS One. 2017 Mar 30;12(3):e0174679. doi: 10.1371/journal.pone.0174679 (PMC5373615; doi:10.1371/journal.pone.0174679)
Supplement: S1 Table — (DOCX) [file pone.0174679.s003.docx]

**S1 Table. Socio-demographic characteristics of individuals in the studied sample (All) and by income quintile (Q1 being the lowest, and Q5 the highest)**

|  | **Income quintiles from the lowest (Q1) to the highest (Q5)** | | | | | | **P** |
| --- | --- | --- | --- | --- | --- | --- | --- |
|  | **All**  (n=1719) | **Q1**  (n=347) | **Q2**  (n=343) | **Q3**  (n=388) | **Q4**  (n=317) | **Q5**  (n=324) |  |
| **Age, y** | 47.0 ± 15.0^1^ | 46.1 ± 14.7 | 47.2 ± 14.7 | 45.5 ± 14.3 | 48.9 ± 16.4 | 47.4 ± 13.6 | <0.001^2^ |
| **Gender, %** |  |  |  |  |  |  | 0.020^3^ |
| Men | 47.6 | 39.5 | 48.5 | 47.1 | 52.9 | 49.6 |  |
| Women | 52.4 | 60.5 | 51.5 | 52.9 | 47.1 | 50.4 |  |
| **Marital status, %** |  |  |  |  |  |  | <0.001^3^ |
| Couple | 74.0 | 63.3 | 79.1 | 74.8 | 77.0 | 75.9 |  |
| Single | 26.0 | 36.7 | 20.9 | 25.2 | 23.0 | 24.1 |  |
| **Number of children, %** |  |  |  |  |  |  | <0.001^3^ |
| Zero | 62.9 | 63.4 | 58.1 | 48.5 | 75.3 | 68.9 |  |
| One at least | 37.1 | 36.6 | 41.9 | 51.5 | 24.7 | 31.1 |  |
| **Educational level^4^, %** |  |  |  |  |  |  | <0.001^3^ |
| Low | 16.7 | 28.0 | 22.0 | 17.3 | 11.1 | 5.3 |  |
| Middle | 51.1 | 53.6 | 55.0 | 55.8 | 51.2 | 38.6 |  |
| High | 32.2 | 18.5 | 23.0 | 26.9 | 37.7 | 56.1 |  |
| **Socio-occupational status^5^, %** |  |  |  |  |  |  | <0.001^3^ |
| Low | 15.6 | 22.5 | 19.8 | 17.4 | 12.8 | 5.5 |  |
| Middle | 34.9 | 29.5 | 34.1 | 40.7 | 33.4 | 36.5 |  |
| High | 9.9 | 2.0 | 5.1 | 8.3 | 8.2 | 27.3 |  |
| Others (retired, students…) | 39.6 | 46.1 | 41.0 | 33.6 | 45.5 | 30.7 |  |
| **Current smoking status, %** |  |  |  |  |  |  | 0.2406^3^ |
| Smoker | 28.4 | 31.4 | 28.7 | 31.8 | 26.3 | 23.5 |  |
| Non-smoker | 71.6 | 68.6 | 71.3 | 68.2 | 73.7 | 76.5 |  |

^1^ Survey weighted mean ± SD (All such values)

^2^ GLM test accounting for survey design was used

^3^ Non-parametric Chi-square test accounting for survey design was used

^4^ The level of education was divided into “high” (university level and equivalent), “intermediate” (high school), and “low” (mid-secondary or under).

^5^ The socio-occupational status divided into “high,” “intermediate”, and “low”. “High” was assigned to executive, top-management and professional classes, “intermediate” to middle professions (office employees, technicians, and similar), and “low” to manual workers and unemployed people. A fourth class, labeled as “others included retired people, students and housewives/house husband
