# Supplementary material for: Dietary changes needed to reach nutritional adequacy without increasing diet cost according to income: An analysis among French adults
Source: PLoS One. 2017 Mar 30;12(3):e0174679. doi: 10.1371/journal.pone.0174679 (PMC5373615; doi:10.1371/journal.pone.0174679)
Supplement: S2 Table — (DOCX) [file pone.0174679.s004.docx]

**S2 Table. Socio-demographic characteristics of individuals in the initial INCA2^1^ adult sample (n=2,624), in the sample of excluded individuals^2^ (n=905) and in the final sample studied (n=1,719).**

|  | **Initial sample**  **(n=2,624)** | **Excluded sample (n=905)** | **Study sample (n=1,719)** | **P** |
| --- | --- | --- | --- | --- |
| **Age, y** | 45.42+16.53^3^ | 42.51 +18.70 | 47.02+15.04 | <0.001^4^ |
|  |  |  |  |  |
| **Gender, %** |  |  |  | 0.195^5^ |
| Men | 48.58 | 50.33 | 47.62 |  |
| Women | 51.42 | 49.67 | 52.38 |  |
|  |  |  |  |  |
| **Income quintiles^6^, %** |  |  |  | 0.003^5^ |
| Q1 | 22.05 | 26.09 | 19.83 |  |
| Q2 | 18.02 | 18.36 | 17.83 |  |
| Q3 | 21.99 | 22.56 | 21.68 |  |
| Q4 | 21.53 | 19.52 | 22.64 |  |
| Q5 | 16.40 | 13.48 | 18.01 |  |
|  |  |  |  |  |
| **Marital status, %** |  |  |  | <0.001^5^ |
| Couple | 68.15 | 57.55 | 73.99 |  |
| Single | 31.85 | 42.45 | 26.01 |  |
|  |  |  |  |  |
| **Number of children, %** |  |  |  | 0.235^5^ |
| Zero | 61.96 | 60.24 | 62.90 |  |
| One at least | 38.04 | 39.76 | 37.10 |  |
|  |  |  |  |  |
| **Educational level, %** |  |  |  | <0.001^3^ |
| Low | 18.44 | 21.61 | 16.69 |  |
| Middle | 52.38 | 54.77 | 51.07 |  |
| High | 29.18 | 23.63 | 32.24 |  |
|  |  |  |  |  |
| **Socio-occupational status, %** |  |  |  | 0.001^5^ |
| Low | 17.77 | 21.63 | 15.64 |  |
| Middle | 34.09 | 32.62 | 34.90 |  |
| High | 8.42 | 5.76 | 9.88 |  |
| Others (retired, students, …) | 39.73 | 39.99 | 39.58 |  |
|  |  |  |  |  |
| **Current smoking status, %** |  |  |  | 0.001^5^ |
| Smoker | 31.24 | 36.42 | 28.41 |  |
| Non-smoker | 68.76 | 63.58 | 71.59 |  |

^1^ The French Individual and National Dietary Survey (INCA2) survey was conducted in 2006–2007 by the ANSES (French Agency for Food, Environmental and Occupational Health Safety) to assess dietary intake and associated behaviours in a nationally representative sample of French people [32]

^2^ individuals aged less than 20y old, or individuals identified as under reporter or individuals with an infeasible mathematical solution for diet modelling

^3^ Survey weighted mean ± SD (All such values)

^4^ GLM test accounting for survey design was used to compare the excluded sample to the study sample

^5^ Non-parametric Chi-square test accounting for survey design was used to compare the excluded sample to the study sample

^6^ quintiles were survey weighted and estimated on the initial sample as follow: 20^th^ = 610, 40^th^ = 940, 60^th^ = 1333, 80^th^ = 1867 euros per month per consumption unit.
